# Supplementary material for: Genomic analysis of morphometric traits in bighorn sheep using the Ovine Infinium® HD SNP BeadChip
Source: PeerJ. 2018 Feb 12;6:e4364. doi: 10.7717/peerj.4364 (PMC5817937; doi:10.7717/peerj.4364)
Supplement: Table S1 — Correlations among females (aged 4 or greater) are below the diagonal, while those for males (aged 4) are above the diagonal. Note that we present the sexes separately given the sexual dimorphism in this species, and these ages as they allow consideration of adult phenotypes (removing autocorrelation with age), while avoiding the effects of selective harvest due to trophy hunting in males as measurements are collected before the hunting season and no male younger than 4 years has ever been legally harvested. [file peerj-06-4364-s001.docx]

Supplementary Table 1: Correlation coefficients among the morphological traits examined in this study. Correlations among females (aged 4 or greater) are below the diagonal, while those for males (aged 4) are above the diagonal. Note that we present the sexes separately given the sexual dimorphism in this species, and these ages as they allow consideration of adult phenotypes (removing autocorrelation with age), while avoiding the effects of selective harvest due to trophy hunting in males as measurements are collected before the hunting season and no male younger than 4 years has ever been legally harvested.

|  | **Horn Length** | **Horn Base Circumference** | **Body Mass** |
| --- | --- | --- | --- |
| **Horn Length** |  | 0.81 | 0.71 |
| **Horn Base Circumference** | 0.29 |  | 0.72 |
| **Body Mass** | 0.28 | 0.23 |  |
