# Supplementary material for: Genomic analysis of morphometric traits in bighorn sheep using the Ovine Infinium® HD SNP BeadChip
Source: PeerJ. 2018 Feb 12;6:e4364. doi: 10.7717/peerj.4364 (PMC5817937; doi:10.7717/peerj.4364)
Supplement: Table S2 — Genotyping was done using the Ovine Infinium® HD SNP BeadChip an array originally developed for domestic sheep containing 606,006 loci. Marker positions were taken from the domestic sheep genome assembly (version 3.1). [file peerj-06-4364-s002.docx]

Supplementary Table 2: Number distribution of markers used in the GWAS analysis. Genotyping was done using the Ovine Infinium® HD SNP BeadChip an array originally developed for domestic sheep containing 606,006 loci. Marker positions were taken from the domestic sheep genome assembly (version 3.1).

| **Chromosome** | **No. Loci** | **Avg. Inter-marker Distance** | **SD of Inter-marker Distance** |
| --- | --- | --- | --- |
| 1 | 429 | 639,475.92 | 722,591.62 |
| 2 | 375 | 662,254.19 | 780,049.65 |
| 3 | 270 | 824,399.96 | 1,012,272.38 |
| 4 | 172 | 685,797.94 | 737,062.79 |
| 5 | 166 | 638,050.85 | 794,621.76 |
| 6 | 142 | 820,292.52 | 961,026.48 |
| 7 | 186 | 527,153.04 | 607,010.96 |
| 8 | 139 | 647,412.07 | 683,785.33 |
| 9 | 129 | 715,575.23 | 924,268.73 |
| 10 | 142 | 605,600.96 | 708,386.17 |
| 11 | 101 | 591,167.02 | 673,572.80 |
| 12 | 134 | 590,221.62 | 789,103.56 |
| 13 | 94 | 880,912.27 | 908,979.65 |
| 14 | 116 | 527,956.87 | 539,733.97 |
| 15 | 170 | 473,971.36 | 588,209.46 |
| 16 | 105 | 656,399.16 | 738,427.39 |
| 17 | 106 | 669,053.35 | 762,647.92 |
| 18 | 110 | 604,523.53 | 619,577.38 |
| 19 | 109 | 556,014.41 | 648,734.61 |
| 20 | 147 | 325,445.54 | 621,916.39 |
| 21 | 63 | 747,203.00 | 782,700.54 |
| 22 | 56 | 841,430.49 | 961,636.08 |
| 23 | 81 | 766,115.53 | 836,916.85 |
| 24 | 73 | 539,960.92 | 651,886.78 |
| 25 | 82 | 541,281.88 | 579,233.56 |
| 26 | 80 | 548,843.14 | 588,078.94 |
